# Autophagy Enhances the Aggressiveness of Human Colorectal Cancer Cells and Their Ability to Adapt to Apoptotic Stimulus

**DOI:** 10.3969/j.issn.2095-3941.2012.02.004

**Published:** 2012-06

**Authors:** Hai-yang Zheng, Xiao-yang Zhang, Xing-fen Wang, Bao-cun Sun

**Affiliations:** 1 Department of Pathology, The Second Hospital of Tianjin Medical University, Tianjin 300211, China; 2 Department of Pathology, Tianjin Medical University Cancer Institute and Hospital, Tianjin 300060, China

**Keywords:** autophagy, apoptosis, colorectal neoplasms, LC3B, caspase-3

## Abstract

**Objective:**

To investigate LC3B-II and active caspase-3 expression in human colorectal cancer to elucidate the role of autophagy, and to explore the relationship of autophagy with apoptosis in human colorectal cancer.

**Methods:**

LC3B expression was detected by immunohistochemistry in 53 human colorectal cancer tissues and 20 normal colon tissues. The protein levels of LC3B-II and active caspase-3 were also determined by Western blot analysis in 23 human colorectal cancer tissues and 10 normal colon tissues.

**Results:**

LC3B was expressed both in cancer cells and normal epithelial cells. LC3B expression in the peripheral area of cancer tissues was correlated with several clinicopathological factors, including tumor differentiation (*P*=0.002), growth pattern of the tumor margin (*P*=0.028), pN (*P*=0.002), pStage (*P*=0.032), as well as vessel and nerve plexus invasion (*P*=0.002). The protein level of LC3B-II in cancer tissue was significantly higher than in normal tissue (*P*=0.038), but the expression of active forms of procaspase-3 in cancer tissue was lower (*P*=0.041). There was a statistically significant positive correlation between the expression levels of LC3B-II and the active forms of procaspase-3 (*r*=0.537, *P*=0.008).

**Conclusions:**

Autophagy has a prosurvival role in human colorectal cancer. Autophagy enhances the aggressiveness of colorectal cancer cells and their ability to adapt to apoptotic stimulus.

## Introduction

Autophagy is responsible for the bulk degradation of intracellular material and is evolutionarily conserved among all eukaryotes ^[^^1^^,^^2^^]^. Autophagy is initiated by formation of the double-membrane vesicle structures (autophagosomes) sequestered from the cytosol and organelles, and then delivered to lysosomes or vacuoles for degradation ^[^^3^^,^^4^^]^. LC3, the mammalian homolog of yeast Atg8, is known to be associated with the autophagosomal membrane ^[^^5^^]^. LC3 is expressed as three isoforms in mammalian cells, namely, LC3A, LC3B, and LC3C. LC3B is first cleaved into the soluble form LC3B-I before it is modified into the membrane-bound form, LC3B-II, and then recruited to autophagosomes. An increase in LC3B-II was shown to be directly correlated with the number of autophagosomes ^[^^6^^]^. LC3B-II is a specific marker of the autophagic process ^[^^7^^,^^8^^]^.

Recent reports indicate that autophagy plays a crucial role in many different physiopathologies, such as during development, nutrient and growth factor deprivation, stress, microbial infection, and diseases ^[^^9^^,^^10^^]^. During these processes, autophagy is induced to recycle cellular constituents and clear damaged organelles to support the bioenergetics of cells and help them adapt to their surroundings. Numerous studies have led to conflicting views on the role of autophagy in tumors. Benign liver adenomas are developed by mice with a systemic mosaic deletion of Atg5, an essential autophagy gene, and liver-specific Atg7^-/-^ mice ^[^^11^^]^. In human breast cancer cells, the ubiquitin-binding protein SQSTM1/p62 and LC3B interact with caspase-8 to promote caspase-8 oligomerization, activation, and apoptosis ^[^^12^^]^. In proapoptotic molecules, p53-deficient HCT116 human colorectal cancer cells combined with the knockdown of all three LC3B isoforms markedly reduce the sub-G1 population, strongly signifying that excessive LC3B levels contribute to enhanced apoptosis ^[^^13^^]^. These studies indicate that autophagy acts as a tumor suppressor. However, other studies yield conflicting results. For example, celecoxib can induce both apoptosis and autophagy in human colorectal cancer cells, and the inhibition of autophagy by pharmacological or genetic means drives colon cancer cells into apoptosis ^[^^14^^]^. Scott et al. ^[^^15^^]^ observed apoptosis in Atg1-overexpressing cells and found that they were due to high levels of autophagy. They concluded that in addition to promoting cell survival, autophagy also promotes cell death when it is induced to high levels ^[^^15^^]^. These studies provide evidence that autophagy serves as a survival pathway in tumor cells.

To date, the role of autophagy as a tumor suppressor or promoter and the relationship of autophagy with apoptosis in colorectal cancerous tissues remain uncertain. This study investigated autophagy and apoptosis activities by assaying the expression of LC3B and caspase-3 in colorectal cancerous tissues using immunohistochemistry and Western blot techniques. The clinicopathologic significance of LC3B and the relationship of autophagy with apoptosis were also explored.

## Patients and Methods

### Patients and samples

Paraffin-embedded samples and fresh tissue specimens were obtained from 53 patients (35 men and 18 women aged 39-84 years) with primary resection of colorectal cancers in the Second Hospital of Tianjin Medical University between January 2009 and May 2010. None of the patients had received neoadjuvant chemotherapy or radiochemotherapy. Informed consent was obtained from all patients according to the guidelines of the local ethics committee.

The study protocol was approved by the ethics committees of the institution. Histological subtyping and tumor grading were performed according to the 2004 World Health Organization classification. Following the methods of Fujii et al. ^[^^16^^]^, cancer tissues were divided into 2 regions, namely, central and peripheral areas. The midpoint between the margin and center of the cancer tissue was defined as the line of demarcation between the peripheral and central areas. The histological margin of a tumor was categorized as pushing or invasive according to the refined criteria proposed by Jass et al.^[^17^]^ An invasive margin was defined as the presence of one or more of the following types of tissue morphology: streaming dissection of muscularis propria, small glands, irregular clusters, cords of cells dissected from mesenteric adipose tissue, or perineurial invasion. Margins not showing these criteria were categorized as pushing ^[^^18^^]^. The pT category, pN category, and pStage of the tumors were determined according to the current TNM classification. Tumor necrosis was defined as cell death in the solid cancer cell nest. All forms of superficial ulcerated necrosis were excluded from this study. The clinicopathologic characteristics of the patients are shown in **Table 1**.

**Table 1:** Relationship of various clinicopathologic factors with LC3B expression in the peripheral area of cancer tissues.

Characteristics	*n*	Peripheral area		*P*	
		Weakly	Strongly			
Age, years					0.972	
﹥70 or ﹤50	21	6	15			
50-70	32	9	23			
Gender						
Male	35	8	27		0.220	
Female	18	7	11			
Tumor site						
Colon	17	6	11		0.653	
Rectum	36	9	27			
Tumor size, cm						
<4.0	14	4	10		1.000	
≥4.0	39	11	28			
Differentiation						
Well-Moderately	28	13	15		0.002	
Poorly	25	2	23			
Margins						
Pushing	15	8	7		0.028	
Invasive	38	7	31			
pT						
pT_1_ or pT_2_	12	3	9		1.000	
pT_3_ or pT_4_	41	12	29			
pN					0.002	
pN_0_	23	10	13			
pN_1_	18	3	15			
pN_2_	12	2	10			
pStage						
I_A_/I_B_/II_A_	23	10	13		0.032	
II_B_/III/IV	30	5	25			
Tumor necrosis						
Yes	10	2	8		0.797	
No	43	13	30			
Vessel and nerve infiltration						
Yes	36	5	31		0.002	
No	17	10	7			

### Methods

#### Immunohistochemistry

For the detection of LC3B expression, rabbit polyclonal antibody against LC3B was used (Cat. No. L7543, Sigma) at a dilution of 1:200 using a PV9000 Kit (Zhongshan Golden Bridge Biotech Co., Ltd.). Tissue sections (4 µm) were obtained from formalin-fixed, paraffin-embedded 53 human colorectal cancer and 20 normal colorectal tissue blocks. All sections were deparaffinized and dehydrated with graded alcohol. Endogenous peroxidase was blocked by incubating the samples for 10 min in 3% H_2_O_2_ at 37°C, heating for 30 min at 95°C to repair antigens, and finally rinsing in PBS. After decanting the PBS, anti-LC3B antibody (1:200) or PBS as a negative control was added and the samples were incubated overnight at 4°C in a humidified chamber. After washing three times in PBS, the slides were treated with anti-rabbit antibody (1:100) for 45 min at 37°C. After complete washing in PBS, the slides were developed in 0.05% freshly prepared diaminobenzidine solution (DAB; Sigma Co.) for 8 min, counterstained with hematoxylin, dehydrated, and mounted on a microscope.

**Figure 1:**
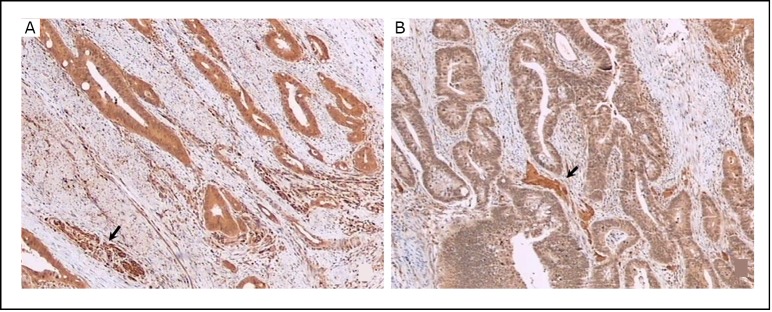
LC3B protein was detected in human colorectal cancer tissues by immunohistochemical staining with LC3B antibody. A: LC3B expression in cancer cells showing equal or stronger intensity than in nerve cells (arrow) were deemed “strongly positive.” B: LC3B expression in cancer cells showing weaker intensity than in nerve cells (arrow) were deemed “weakly positive”. Original magnification, × 40.

The nerve plexus cells in the submucosal or muscularis portions of the colorectal wall showed constantly intensive staining for LC3B ^[^^19^^]^. Thus, each section had its own positive internal control. On this basis, LC3B expression in tumor cells or in normal epithelia was compared with that in nerve cells of the same sections. The following subgroups of nerve cells were then identified: *i*) those with similar or enhanced LC3B expression in tumor cells or normal epithelia compared with those in nerve cells were judged as ‘‘strongly positive” (**Figure 1A**), and *ii*) those with weaker LC3B expression in tumor cells or normal epithelia than those in nerve cells were designated as ‘‘weakly positive’’ (**Figure 1B**). All immunostaining experiments were evaluated twice by 2 pathologists blinded to the patient outcome and other clinical findings.

#### Western blot analysis

Total protein was extracted from tissue samples using lysis buffer [1% SDS, 10 mmol/L Tris-HCl (pH 7.4), and 1 mmol/L Na_3_VO_4_]. The protein concentrations were measured by the BCA protein assay. About 10 µg of protein samples were loaded onto 15% SDS-PAGE gels. The separated proteins were then electrophoretically transferred onto a polyvinylidene difluoride membrane, which was blocked with 5% non-fat dried milk in Tris-buffered saline-Tween (TBST) to block nonspecific binding sites. The blots were then incubated overnight at 4°C with anti-LC3B antibody (No. L7543, Sigma; 1/4000 dilution in TBST and 0.01 g/mL BSA) and anti-caspase-3 antibody (Zhongshan Golden Bridge Biotech Co., Ltd; 1/1000 dilution in TBST and 0.01 g/mL BSA). After extensive washing, polyclonal anti-rabbit horseradish peroxidase-conjugated secondary antibody (1/5000 dilution in TBST and 0.01 g/mL BSA) was added to the membranes for 1 h at room temperature. Signals were visualized using enhanced chemiluminescence detection reagents, and the blots were quantified using Image J software (National Institutes of Health). β-actin was used as a control. The protein levels of LC3B-II, procaspase-3, and its active fragment caspase-3 were normalized to β-actin.

### Statistical analysis

Statistical analysis was performed using the SPSS package (version 16.0; Chicago, IL, USA). The Chi-square test was used to analyze the relationship of LC3B expression in tumors with clinicopathologic parameters. Student’s *t*-test was performed to compare the protein levels of LC3B-II or active caspase-3 in colorectal cancerous tissues with those in normal tissues. The correlations between the protein levels of LC3B-II and caspase-3 active fragment in cancer tissue were assessed by Spearman’s correlation analysis. Two-tailed *P* values of 0.05 or less were considered statistically significant.

## Results

### LC3B expression in colorectal cancer tissue

**Figure 2:**
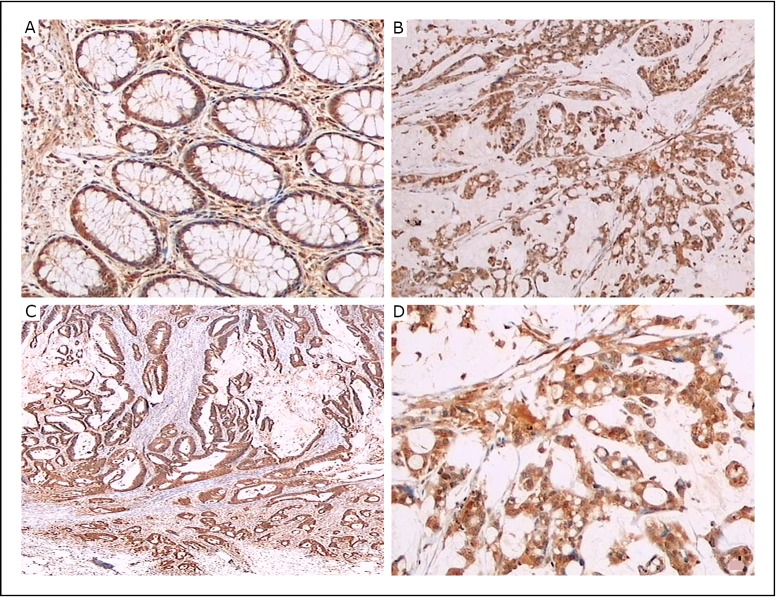
LC3B protein was detected in human colorectal cancer tissues and normal tissues by immunohistochemical staining with LC3B antibody. A: Columnar epithelial cells of normal colon mucosa expressing LC3B; original magnification, × 40. B, D: LC3B expression in poorly differentiated cancer; original magnification, × 20 and × 40. C: LC3B expression in moderately differentiated cancer; original magnification, × 20.

**Figure 3:**
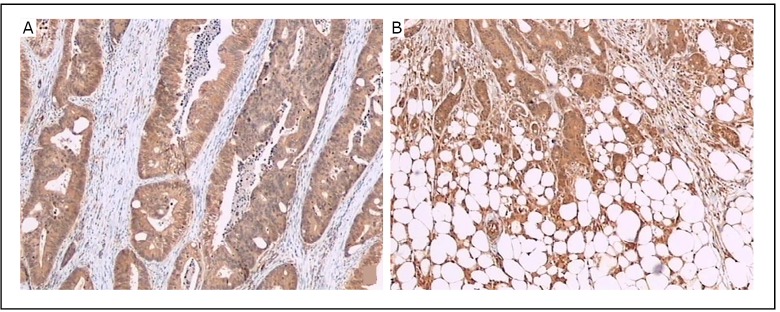
LC3B protein was detected in human colorectal cancer tissues by immunohistochemical staining with LC3B antibody. The intensity of LC3B expression is stronger in the peripheral area (B) than in the central area (A) in the same tissue. Original magnification, × 40.

Almost all 53 primary colorectal cancer specimens expressed LC3B as revealed by immunohistochemistry with anti-LC3B antibody (**Figure 2**). However, LC3B expression in a tumor specimen was usually heterogeneous, with the expression in the peripheral area of the cancer tissue (**Figure 3**) stronger than in the central area (**Figure 3**) in most cases. Thus, the cancer tissues were divided into central and peripheral areas. An analysis was performed to explore the relationships of the levels of LC3B expression (strongly positive or weakly positive) in the central or peripheral area of the cancer tissues with the clinicopathologic factors, including age, gender, tumor site, tumor size, differentiation, tumor margin, pT, pN, pStage, tumor necrosis, as well as vessel and nerve plexus invasion. The intensity level of LC3B expression in the peripheral area of the tumor was significantly correlated with the differentiation (*P*=0.002), tumor margin (*P*=0.028), pN (*P*=0.002), pStage (*P*=0.032), as well as vessel and nerve plexus invasion (*P*=0.002) (**Table 1**). However, the intensity level of LC3B expression in the central area was not correlated with these clinicopathologic factors (data not shown).

### LC3B expression in normal colon mucosa

LC3B expression was observed in normal colon mucosa (**Figure 2A**). LC3B was expressed in the cytoplasm and/or nuclei of the columnar epithelial cells of the crypts. The mucus-containing apical parts of the columnar cells were not stained. Some infiltrating mononuclear cells in the stroma, nerve plexus cells in the submucosal or muscularis regions, and submucosal lymph follicles showed particularly intensive staining (not shown).

### LC3B-II and active caspase-3 expression detected by Western blot

**Figure 4:**
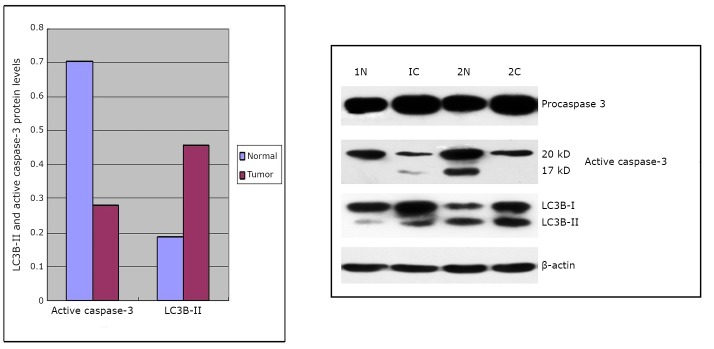
Expression of LC3B-II, procaspase-3, and active forms of caspase-3 in normal tissues (1N, 2N) and cancer tissues (1C, 2C) detected by Western blot analysis.

The expression of LC3B-II and active caspase-3 in 23 human colorectal cancer tissues and 10 normal colorectal tissues was detected by Western blot analysis. As shown in **Figure 4**, LC3B-II, procaspase-3, and its corresponding active form caspase-3 were observed in normal colon and cancer tissues. The Western blot showed that the protein levels of LC3B-II in cancer tissue were significantly higher than those in normal tissue (*P*=0.038). The active forms of procaspase-3 were markedly reduced in cancer tissue than in normal tissue (*P*=0.041). Analysis of the correlation of LC3B-II with the active forms of caspase-3 indicated a statistically significant positive correlation between the expression of LC3B-II and the active forms of caspase-3 (*r*=0.537, *P*=0.008).

## Discussion

Autophagy can be classified into basal and induced modes^[^^20^^]^. In normal cells, the basal levels of autophagy maintain homeostasis by eliminating excessive or unnecessary proteins and injured or aged organelles. On the other hand, autophagy is observed under some pathological conditions, including some aggressive malignant tumors ^[^^9^^,^^10^^]^ in which cancer cells are likely to encounter a shortage of nutrients. Both basal and induced levels of autophagy are important in human health and disease.

Autophagy contributes to the survival of established tumor cells under various stress conditions. In general, cells in the interior part of tumor, where nutrients and oxygen are less abundant than in outer regions, tend to show higher levels of autophagy than cells at the tumor margins ^[^^21^^]^. In the present study, LC3B was expressed in nearly all cancer cells, consistent with the results of other studies ^[^19,22^]^. However, LC3B expression within a tumor specimen was heterogenous, with the stronger expression in the peripheral area of the cancer tissue than in the central area in most cases, especially in invasive margins. Thus, the staining intensity of LC3B in both peripheral and central areas was evaluated. This study found a significant correlation between LC3B expression and tumor aggressiveness, such as tumor differentiation, tumor margin, pN, pStage, as well as vessel and nerve plexus invasion. However, LC3B expression in the central area was found to be unrelated to these clinicopathologic factors. These results indicate that the excessive upregulation of autophagy can not only maintain tumor cell survival, but also promote their aggressiveness. In contrast to previous studies ^[^^19^^,^^22^^,^^23^^]^, this study found LC3B expression in some noncancerous epithelial cells, consistent with the basal function of autophagy. Although counting autophagic vesicles by electron microscopy is recognized as the standard method for assessing autophagic activity ^[^^6^^,^^7^^,^^24^^]^, it is not capable of evaluating entire tumor samples. Immunohistochemical staining is a useful method for evaluating autophagic activity in surgically resected cancer specimens, and it has also been adopted by many studies ^[^^16^^,^^19^^,^^22^^]^.

Recently, several groups of investigators observed the interaction between autophagy and apoptosis by altering the molecules associated with these processes ^[^^25^^]^. The inhibition of autophagy by the vacuolar type H^+^-ATPase inhibitor bafilomycin A1 reportedly lowers G_1_-S transition and induces apoptosis in colon cancer cells ^[^^26^^]^. Both autolysosome inhibitors and 3-MA induce the marked apoptotic death of all examined colorectal cancer cells ^[^^27^^]^. Most studies on the correlation between autophagy and apoptosis were performed *in vitro* using cell lines. However, the human body is extremely complex and these *in vitro* observations cannot completely represent a variety of actual *in vivo* situations. The protein levels of LC3B, including LC3B-I and LC3B-II, are evaluated by immunohistochemical staining. However, monitoring LC3B-II expression by Western blot analysis is essential and possibly the most reliable method for detecting autophagy ^[^^6^^,^^7^^,^^24^^]^. During apoptosis, caspase cascades are activated and play a central role in the execution of apoptosis. Caspase-3 is synthesized as an inactive proenzyme that is then activated by cleavage in cells undergoing apoptosis^[^^28^^]^. The use of activated caspase-3 antibody staining to indicate apoptosis in breast cancer was validated ^[^^29^^]^. The present study also found a statistically significant positive correlation between the levels of LC3B-II and active caspase-3 in human colorectal cancer tissues. Combinied with immunohistochemistry staining results, it can be presumed that autophagy may be an adaptive response that allows cancer cells to survive an apoptotic stimulus. When cancer cells encounter stresses that can induce apoptosis, autophagy is activated not only to provide nutrients and oxygen by recycling cellular constituents to maintain cell survival, but also to clear accumulated damaged organelles and protect against cell death. Thus, the targeted inhibition of autophagy may be a useful strategy for colorectal cancer treatment. Further investigations with larger sample sizes are required to get full understanding of the contributions of autophagy to colorectal cancer development as well as identify other oncogenes and tumor suppressor genes for determining the outcome of combined autophagy inhibition and apoptosis induction.

The current data suggest that autophagy is activated in human colorectal cancer cells. Autophagy preferentially protects tumor cells in the peripheral area of cancer tissues, and enhances their aggressiveness and ability to adapt to apoptotic stimulus. Therefore, autophagy performs a prosurvival role in colorectal cancer cells.
